# Left ventricular dysfunction with reduced functional cardiac reserve in diabetic and non-diabetic LDL-receptor deficient apolipoprotein B100-only mice

**DOI:** 10.1186/1475-2840-10-59

**Published:** 2011-06-30

**Authors:** Suvi E Heinonen, Mari Merentie, Marja Hedman, Petri I Mäkinen, Elina Loponen, Ivana Kholová, Fatima Bosch, Markku Laakso, Seppo Ylä-Herttuala

**Affiliations:** 1 Department of Biotechnology and Molecular Medicine at A.I. Virtanen Institute for Molecular Sciences, University of Eastern Finland, P.O. Box 1627, FI-70211 Kuopio, Finland; 2 Heart Center, Kuopio University Hospital, P.O. Box 1777, FI-70211 Kuopio, Finland; 3 Pathology, Laboratory Centre, Tampere University Hospital, P.O. Box 2000, FI-33521 Tampere, Finland; 4 Center of Animal Biotechnology and Gene Therapy, Universitat Autònoma de Barcelona, E-08193 Bellaterra, Spain; 5 Department of Medicine, University of Eastern Finland and Kuopio University Hospital, FI-70210, Kuopio, Finland

## Abstract

**Background:**

Lack of suitable mouse models has hindered the studying of diabetic macrovascular complications. We examined the effects of type 2 diabetes on coronary artery disease and cardiac function in hypercholesterolemic low-density lipoprotein receptor-deficient apolipoprotein B100-only mice (LDLR^-/-^ApoB^100/100^).

**Methods and results:**

18-month-old LDLR^-/-^ApoB^100/100 ^(n = 12), diabetic LDLR^-/-^ApoB^100/100 ^mice overexpressing insulin-like growth factor-II (IGF-II) in pancreatic beta cells (IGF-II/LDLR^-/-^ApoB^100/100^, n = 14) and age-matched C57Bl/6 mice (n = 15) were studied after three months of high-fat Western diet. Compared to LDLR^-/-^ApoB^100/100 ^mice, diabetic IGF-II/LDLR^-/-^ApoB^100/100 ^mice demonstrated more calcified atherosclerotic lesions in aorta. However, compensatory vascular enlargement was similar in both diabetic and non-diabetic mice with equal atherosclerosis (cross-sectional lesion area ~60%) and consequently the lumen area was preserved. In coronary arteries, both hypercholesterolemic models showed significant stenosis (~80%) despite positive remodeling. Echocardiography revealed severe left ventricular systolic dysfunction and anteroapical akinesia in both LDLR^-/-^ApoB^100/100 ^and IGF-II/LDLR^-/-^ApoB^100/100 ^mice. Myocardial scarring was not detected, cardiac reserve after dobutamine challenge was preserved and ultrasructural changes revealed ischemic yet viable myocardium, which together with coronary artery stenosis and slightly impaired myocardial perfusion suggest myocardial hibernation resulting from chronic hypoperfusion.

**Conclusions:**

LDLR^-/-^ApoB^100/100 ^mice develop significant coronary atherosclerosis, severe left ventricular dysfunction with preserved but diminished cardiac reserve and signs of chronic myocardial hibernation. However, the cardiac outcome is not worsened by type 2 diabetes, despite more advanced aortic atherosclerosis in diabetic animals.

## Background

Type 2 diabetes is associated with a marked increase in the risk of coronary heart disease [1,2]. Still, the mechanisms underlying macrovascular diseases in type 2 diabetic patients remain unclear. In recent years various models combining disorders of lipid and glucose metabolism have been generated and they have provided valuable information. However, better and more appropriate models are urgently needed.

To date, development of mouse models describing diabetic macrovascular disease has been limited. The effect of hyperglycemia has been widely studied in atherosclerotic models by streptozotosin-induced beta cell destruction [3]. However, while hyperglycemia is an important factor in microvascular complications of diabetes, its role in macrovascular events is less dominant [4] and animal studies have not been consistent [5-8]. Thus, achieving models representing the overall metabolic changes typical for diabetic patients with the highest risk of macrovascular complications has mostly been pursued by cross-breeding leptin-deficient (ob/ob) or leptin receptor-deficient (db/db) mice with atherosclerotic ApoE^-/- ^[9-11] and LDLR^-/- ^[9,12] mice. Recently the ob/ob mouse was cross-bred also with ApoE^-/-^ApoB^100/100 ^and LDLR^-/-^ApoB^100/100 ^mice [13], resulting in promising models of metabolic syndrome. However, a major shortcoming in these models is that the perturbation of glucose metabolism does not appear to affect atherosclerosis without concomitant elevations in plasma lipid levels [14,15]. Also data about their cardiac phenotype is scarce - there are only a few studies on ventricular function of ob/ob/LDLR^-/- ^mice [16-18], although differentiating the effects of diabetes from those of hypercholesterolemia cannot be done without comparisons to non-diabetic hypercholesterolemic mice. In addition, the status of coronary artery atherosclerosis and its relation to cardiac function have not been studied in any comparable models of diabetic macrovascular disease.

We have previously developed a mouse model of diabetic macroangiopathy, where mice overexpressing IGF-II in pancreatic beta cells [19] in hypercholesterolemic LDLR^-/-^ApoB^100/100 ^background represent hyperglycemia, insulin resistance and mild hyperinsulinemia without concomitant changes in plasma lipid levels [20]. To study the cardiac phenotype of hypercholesterolemic LDLR^-/-^ApoB^100/100 ^mice and to see if it is impaired by diabetes, we now evaluated the extent of coronary artery disease (CAD) and its effects on myocardial perfusion, left ventricular function and cardiac reserve *in vivo*. Senescent animals were used to simulate the respective human patient population, and C57Bl/6 mice were included to control the effects of aging and high fat diet.

## Methods

### Animals

We have previously developed IGF-II/LDLR^-/-^ApoB^100/100 ^mouse as a model of diabetic macroangiopathy [20]. In the present study, IGF-II negative LDLR^-/-^ApoB^100/100 ^littermates served as controls and we also included matched C57Bl/6J mice to control the effects of aging and high-fat diet. Mice (n = 12-15/strain) were fed *ad libitum *with a normal chow diet (R36, Lactamin, Sweden). At the age of 15 months mice were put on a high fat Western diet (TD 88173, Harlan Teklad: 42% of calories from fat and 0.15% from cholesterol) for three months, thus the animals were 18 months old at the time of analysis. Both female and male mice were used in this study. Experiments were approved by the National Experimental Animal Board.

### Metabolic analyses

Glucose tolerance test (GTT) and blood glucose measurements were carried out in mice fasted overnight (15 h) as described [20]. Triglycerides and total cholesterol were determined from fasting plasma samples as described [21].

### Echocardiography

Transthoracic echocardiography was performed in isoflurane anesthetized mice at rest and after inotropic stimulation with dobutamine using a high-resolution imaging system for small animals (Vevo 770, VisualSonics Inc., Canada) equipped with a high-frequence ultrasound probe (RMV-707B) as described [22]. Left ventricular (LV) dimensions and wall thicknesses were determined from parasternal short axis M-mode images. Ejection fraction (EF), fractional shortening (FS), LV volume and LV mass were calculated by Vevo770 software. EF was determined by using the Teicholz formula. The data represent averaged values of 3 cardiac cycles. The β-adrenoceptor agonist dobutamine (1 μg/g) was injected intraperitoneally (n = 5-6/strain) and echocardiography was performed at baseline and 1, 5, 10 and 15 minutes after injection. The values of individual peak response were used. Regional LV wall motion was evaluated before and after dobutamine stress from electrocardiogram kilohertz-based visualization (EKV™) cine loops. A 12-segment model on two levels of short axis views (mid-ventricle and apical) comprising of 6 wall segments (anterior, anterolateral, inferolateral, inferior, and inferoseptal) was used. Wall motion was scored 1 = normal, 2 = hypokinetic, 3 = akinetic and 4 = dyskinetic, and a score index was calculated as the sum of scores divided by the total number of segments. In addition to the regional evaluation of the myocardium in dobutamine stress echocardiography, LV wall motion was also assessed in all animals (n = 11-13/strain) from long-axis cine view.

### Myocardial perfusion

Myocardial perfusion was quantified as described [22]. Briefly, perfusion was measured with Cadence™ contrast pulse sequence (CPS) ultrasound by Acuson Sequoia C256 using 15L8 probe (Siemens Medical Solutions, USA) and a second generation microbubble contrast agent (SonoVue, Bracco Diagnostics Inc., USA) administered as a 50 μl bolus via tail vein. Datapro (v2.13, Noesis SA, France) was used to quantify CPS signal intensities.

### Histology

Mice were sacrificed with carbon dioxide and perfused with phosphate-buffered saline. Tissue samples were immersion-fixed with 4% paraformaldehyde (pH 7.4). Aortas were embedded in paraffin after which cross-sectional lesion areas from the sinus level [21] and lesion calcification [20] were quantified as described. To quantify coronary artery atherosclerosis the maximal luminal narrowing was measured as a percentage from the area enclosed by the internal elastic lamina in serial cross-sections [23]. Vascular remodeling ratio was calculated by dividing individual cross-sectional vessel areas by the average area of the C57Bl/6J controls, and classified as inward (< 0.95), absent (0.95-1.05) or outward remodeling (> 1.05) [24]. Myocardial fibrosis was quantified from Masson Trichome stained serial sections from the level of basal ventricle to apex. Analyses were performed in a blinded fashion using AnalySIS software (Soft Imaging System GmbH).

### Electron microscopy

Tissue specimens were fixed overnight in 2.5% glutaraldehyde, treated with 1% osmium tetroxide, dehydrated and embedded in LX-112 resin and polymerized. The 60-70 nm sections were contrasted with uranyl acetate and lead citrate and examined by transmission electron microscopy (JEM 1200EX, JEOL Ltd. Japan).

### Statistical analysis

Student's paired t-test was used for analyzing individual response to dobutamine stress, one-way ANOVA with Bonferroni's post test was used for detecting differences between the mouse strains and ANOVA of repeated measures was applied to analyze the results of GTT. P < 0.05 was considered significant. Numerical values are shown as mean ± standard deviation (SD). Statistical analyses were performed using GraphPad Prism 4.0.

## Results

### Basic metabolic parameters

**Figure 1:**
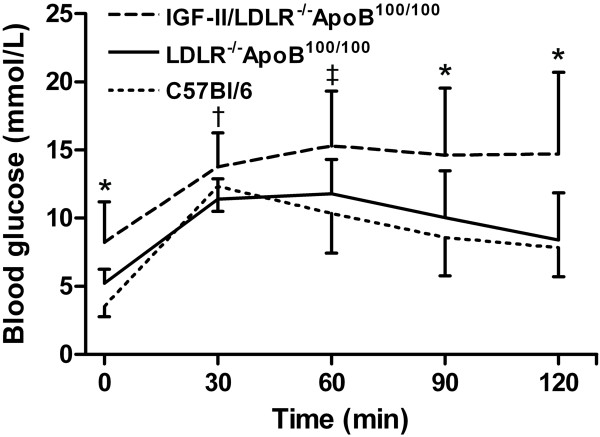
**IGF-II/LDLR^-/-^ApoB^100/100 ^mice show elevated fasting glucose and glucose intolerance in an intraperitoneal GTT**. Values denote mean ± SD of 9 (LDLR^-/-^ApoB^100/100^) or 12 (C57Bl/6J and IGF-II/LDLR^-/-^ApoB^100/100^) 18-month-old mice. * *P *< 0.05 in IGF-II/LDLR^-/-^ApoB^100/100 ^versus C57Bl/6J and LDLR^-/-^ApoB^100/100 ^mice, † *P *< 0.05 between IGF-II/LDLR^-/-^ApoB^100/100 ^and LDLR^-/-^ApoB^100/100 ^mice, ‡ *P *< 0.05 between IGF-II/LDLR^-/-^ApoB^100/100 ^and C57Bl/6J mice.

Metabolic parameters are presented in Table 1. Interestingly, body weight tended to be higher in C57Bl/6J mice, especially in males. Nevertheless, it did not reflect on other metabolic factors such as blood glucose or glucose tolerance - the IGF-II/LDLR^-/-^ApoB^100/100 ^mice showed both fasting hyperglycemia (8.2 ± 3.0 mmol/l) and impaired glucose tolerance in intraperitoneal GTT (2-hour post-glucose load 14.7 ± 6.0 mmol/l) compared to LDLR^-/-^ApoB^100/100 ^and C57Bl/6J mice (Table 1 Figure 1). Due to common genetic background and high fat diet the total cholesterol levels were significantly higher, although equal, in both LDLR^-/-^ApoB^100/100 ^and IGF-II/LDLR^-/-^ApoB^100/100 ^mice (25.5 ± 7.9 and 27.8 ± 5.0 mmol/l, respectively) compared to C57Bl/6J controls (3.9 ± 1.4 mmol/l). However, in triglycerides there was no difference between C57Bl/6J and LDLR^-/-^ApoB^100/100 ^mice (1.0 ± 0.3 and 1.1 ± 0.3 mmol/l, respectively), whereas in diabetic IGF-II/LDLR^-/-^ApoB^100/100 ^mice the concentration was slightly elevated (1.5 ± 0.5 mmol/l, *P *< 0.05).

**Table 1:** Metabolic parameters, echocardiographic measurements and myocardial perfusion.

	C57Bl/6J	LDLR^-/-^ApoB^100/100^	IGF-II/LDLR^-/-^ApoB^100/100^
**Metabolic parameters**			

Body weight (g)			

Females	34.4 ± 6.5	28.8 ± 1.9	33.3 ± 3.1

Males	45.8 ± 5.6^‡^	35.4 ± 4.2*^‡^	36.8 ± 3.7

Glucose (mmol/l)	3.5 ± 0.8	5.2 ± 1.0	8.2 ± 3.0*^†^

Cholesterol (mmol/l)	3.9 ± 1.4	25.5 ± 7.9*	27.8 ± 5.0*

Triglycerides (mmol/l)	1.0 ± 0.3	1.1 ± 0.3	1.5 ± 0.5*^†^

**Echocardiographic measurements in baseline**			

HR (bpm)	443.5 ± 41.0	504.8 ± 14.1*	486.1 ± 30.1*

Dimensions			

LVID_ED _(mm)	3.45 ± 0.38	4.13 ± 0.61*	4.09 ± 0.38*

LVID_ES _(mm)	2.3 ± 0.44	3.25 ± 0.80*	3.14 ± 0.51*

LVPW;d (mm)	1.26 ± 0.27	0.88 ± 0.18*	0.90 ± 0.18*

LVPW;s (mm)	1.70 ± 0.34	1.13 ± 0.24*	1.15 ± 0.26*

LVAW;d (mm)	1.22 ± 0.17	0.95 ± 0.17*	1.04 ± 0.13*

LVAW;s (mm)	1.77 ± 0.22	1.18 ± 0.25*	1.39 ± 0.22*

LV Vol;d (μl)	49.88 ± 12.34	77.88 ± 26.49*	71.11 ± 12.43*

LV Vol;s (μl)	14.22 ± 7.32	46.23 ± 27.07*	37.59 ± 13.37*

LV Mass/Body weight	4.42 ± 0.69	4.73 ± 1.58	4.10 ± 0.78

Systolic function			

EF (%)	72.98 ± 9.45	44.86 ± 14.70*	48.17 ± 11.06*

FS (%)	41.74 ± 7.72	22.46 ± 8.23*	26.83 ± 10.73*

**Myocardial perfusion** (ratio to C57Bl/6J)			

LVAW	1.00 ± 0.06	0.97 ± 0.09	0.93 ± 0.19

Values denote mean ± SD of 12-15 C57Bl/6J mice, 9-12 LDLR^-/-^ApoB^100/100 ^mice and 10-14 IGF-II/LDLR^-/-^ApoB^100/100 ^mice. Metabolic parameters represent values after overnight fasting. Glucose was measured from whole blood. Cholesterol and triglycerides were analyzed from plasma. Cardiac measurements were obtained from echocardiography. Perfusion was measured with contrast pulse sequence ultrasound. HR, heart rate; LV, left ventricle; ID, internal diameter; ED, end-diastole; ES, end-systole; s, systole; d, diastole; PW, posterior wall; AW, anterior wall; Vol, volume; EF, ejection fraction; FS, fractional shortening.

*P < 0.05 vs. C57Bl/6J

† *P *< 0.05 vs. LDLR^-/-^ApoB^100/100^

‡ *P *< 0.05 vs. females of the same strain

### Atherosclerosis in hypercholesterolemic mice is accompanied by compensatory vascular remodeling and aggravated by calcification in IGF-II/LDLR^-/-^ApoB^100/100 ^mice

**Figure 2:**
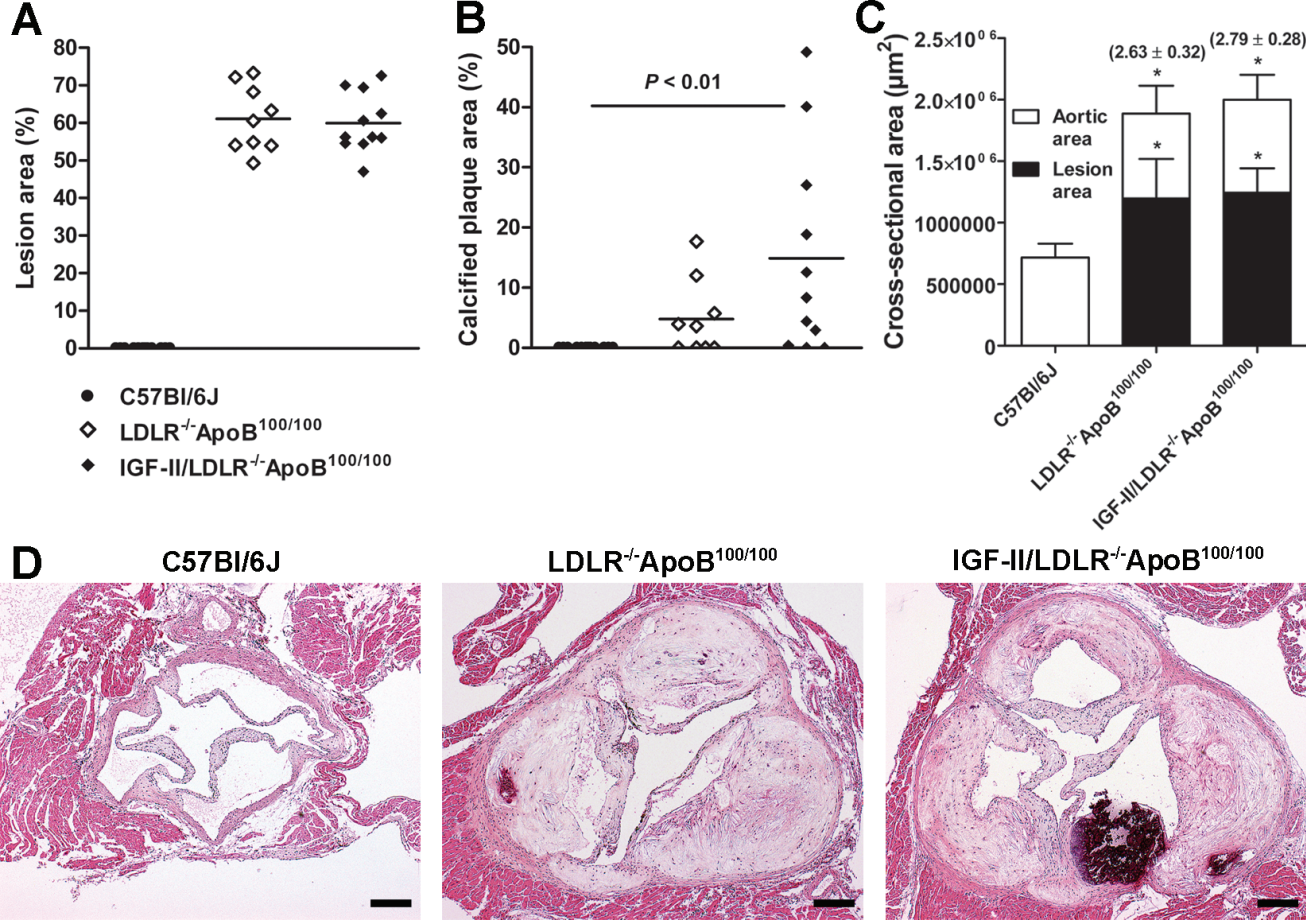
**Evaluation of aortic atherosclerosis and vascular remodeling**. A) Cross-sectional lesion areas from the aortic sinus level, B) calcification of atherosclerotic plaques and C) average absolute vessel (white bars) and lesion areas (black bars) in the aortic sinus (mean ± SD) of 18-month-old mice. In comparison to C57Bl/6J controls (n = 8), compensatory enlargement of vessel area is seen in LDLR^-/-^ApoB^100/100 ^(n = 9) and IGF-II/LDLR^-/-^ApoB^100/100 ^mice (n = 11) with vascular remodeling ratios in parenthesis indicating positive remodeling (> 1.05) in response to atherosclerosis. * *P *< 0.05 versus C57Bl/6J. D) Representative hematoxylin-eosin stained aortic sections from sinus level of C57Bl/6J (left panel), LDLR^-/-^ApoB^100/100 ^(middle) and IGF-II/LDLR^-/-^ApoB^100/100 ^mice (right panel). Original magnification × 40, scale bar 100 μm.

Atherosclerosis was quantified from serial aortic cross-sections from the sinus level. No lesions were detected in aortas of C57Bl/6J mice, whereas in LDLR^-/-^ApoB^100/100 ^and IGF-II/LDLR^-/-^ApoB^100/100 ^mice lesion areas were 61.1 ± 8.7% and 60.0 ± 7.9% of the total aortic area, respectively (Figure 2A, D). Although the diabetic background did not affect lesion size per se, there was more intimal calcification in IGF-II/LDLR^-/-^ApoB^100/100 ^mice (18.2 ± 17.3% of plaque area) compared to hypercholesterolemic LDLR^-/-^ApoB^100/100 ^mice (8.6 ± 6.1% of plaque area) or C57Bl/6J mice (no calcification detected) (Figure 2B, D). Atherosclerosis was associated with compensatory enlargement of the vessel area with a vascular remodeling ratio of 2.63 ± 0.32 in LDLR^-/-^ApoB^100/100 ^and 2.79 ± 0.28 in IGF-II/LDLR^-/-^ApoB^100/100 ^mice compared to C57Bl/6J mice (Figure 2C). Consequently, despite extensive atherosclerosis, lumen areas were not decreased in LDLR^-/-^ApoB^100/100 ^(690.3 ± 201.2 × 10^3 ^μm^2^) or IGF-II/LDLR^-/-^ApoB^100/100 ^mice (756.4 ± 149.9 × 10^3 ^μm^2^) compared to non-atherosclerotic C57Bl/6J controls (716.4 ± 112.9 × 10^3 ^μm^2^).

### Severe coronary artery stenosis in hypercholesterolemic mice

**Figure 3:**
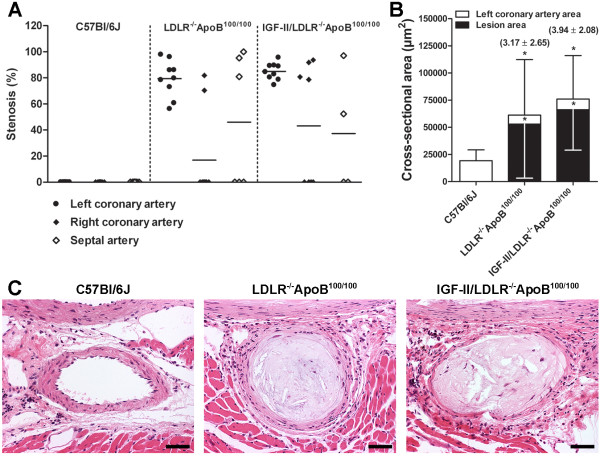
**Analysis of coronary artery atherosclerosis and remodeling**. A) The degree of luminal stenosis in coronary arteries and B) average absolute vessel (white bars) and respective lesion areas (black bars) in the left coronary artery of 18-month-old mice (mean ± SD). In comparison to C57Bl/6J controls (n = 9), compensatory enlargement of the left coronary artery is seen in LDLR^-/-^ApoB^100/100 ^(n = 7) and IGF-II/LDLR^-/-^ApoB^100/100 ^mice (n = 7) with vascular remodeling ratios in parenthesis indicating positive remodeling (> 1.05) in response to atherosclerosis. * *P *< 0.05 versus C57Bl/6J. C) Representative hematoxylin-eosin stained sections showing severe stenosis in proximal left coronary artery in LDLR^-/-^ApoB^100/100 ^(middle) and IGF-II/LDLR^-/-^ApoB^100/100 ^mice (right panel). Original magnification × 200, scale bar 25 μm.

Significant stenosis was observed in ostial parts of coronary arteries in LDLR^-/-^ApoB^100/100 ^and IGF-II/LDLR^-/-^ApoB^100/100 ^mice. The stenosis was most prominent in the left coronary artery, which was narrowed in all studied LDLR^-/-^ApoB^100/100 ^and IGF-II/LDLR^-/-^ApoB^100/100 ^mice with average luminal stenosis of 79.4 ± 14.3% and 85.0 ± 6.5%, respectively (Figure 3A, C). This was accompanied with outward remodeling: compared to C57Bl/6J, remodeling ratio of the left coronary artery was 3.17 ± 2.65 in LDLR^-/-^ApoB^100/100 ^and 3.94 ± 2.08 in IGF-II/LDLR^-/-^ApoB^100/100 ^mice (Figure 3B). However, in spite of the vessel enlargement, significant decrease of the left coronary artery lumen area was present in both LDLR^-/-^ApoB^100/100 ^(8.3 ± 5.8 × 10^3 ^μm^2^, *P *< 0.01) and IGF-II/LDLR^-/-^ApoB^100/100 ^mice (9.8 ± 4.9 × 10^3 ^μm^2^, *P *< 0.05) compared to C57Bl/6J controls (19.3 ± 10.0 × 10^3 ^μm^2^). In the right coronary artery, 22% of LDLR^-/-^ApoB^100/100 ^and 50% of IGF-II/LDLR^-/-^ApoB^100/100 ^mice showed mean stenosis of 76.1 ± 8.2% and 86.2 ± 7.6%, respectively (Figure 3A). Stenosis of the septal artery was detected in 50% of mice in both groups with average stenosis of 92.0 ± 10.0% in LDLR^-/-^ApoB^100/100 ^and 74.7 ± 31.6% in IGF-II/LDLR^-/-^ApoB^100/100 ^mice (Figure 3A). Despite severe atherosclerosis in the proximal coronary arteries, no lesions were detected in the distal parts. In C57Bl/6J mice no lesion formation was observed in any part of the coronary tree.

### Coronary atherosclerosis leads to left ventricular dysfunction

Echocardiography was performed to examine *in vivo *LV function. LV chamber dimensions, anterior wall (AW) and posterior wall (PW) thicknesses and LV mass were obtained from M-mode images (Table 1). Compared to C57Bl/6J mice, LV end-diastolic and end-systolic diameters were significantly increased in both LDLR^-/-^ApoB^100/100 ^and IGF-II/LDLR^-/-^ApoB^100/100 ^mice. LV dilatation was demonstrated by increased LV end-diastolic and end-systolic volumes and dilatation was most pronounced in LDLR^-/-^ApoB^100/100 ^mice. Related to LV dilatation, reduced thickness of both posterior and anterior LV wall was evident in both LDLR^-/-^ApoB^100/100 ^and IGF-II/LDLR^-/-^ApoB^100/100 ^mice. In line with the impaired cardiac function, resting heart rate was higher in LDLR^-/-^ApoB^100/100 ^and IGF-II/LDLR^-/-^ApoB^100/100 ^mice compared to C57Bl/6J controls.

LV systolic function was evaluated with the Teicholz method of the EF and FS in M-mode images. Both were significantly reduced in LDLR^-/-^ApoB^100/100 ^and IGF-II/LDLR^-/-^ApoB^100/100 ^mice depicting severe contractile dysfunction, whereas C57Bl/6J controls showed normal cardiac function (Table 1). In visual evaluation of the parasternal long axis 2-dimensional EKV™ cine loops [see Additional files 1, 2 &3], LVAWs of the C57Bl/6J controls were mostly normokinetic with only mild hypokinesia noted in two animals and a dyskinetic area in one animal. Impaired wall motion with hypokinesia was common in LDLR^-/-^ApoB^100/100 ^(in 10/13 mice, 76.9%) and IGF-II/LDLR^-/-^ApoB^100/100 ^mice (in 8/11 mice, 72.7%), and akinetic areas were observed in altogether four animals: one LDLR^-/-^ApoB^100/100 ^and three IGF-II/LDLR^-/-^ApoB^100/100 ^mice. Animals with severe LV wall dysfunction had high aortic lesion area (> 70%), calcified plaques (4 - 12% of plaque area), severe coronary artery stenosis (> 93% occlusion in left coronary artery) and LV ejection fraction of 18 - 41%. Despite of the detected akinesia in the echocardiograms there were neither increased echogenicity of the affected area, nor histological observations of increased fibrosis referring to myocardial infarction scar in Masson-Trichrome stained serial sections (data not shown). Moreover, although the overall mortality did not differ between mouse models, one animal in both LDLR^-/-^ApoB^100/100 ^and IGF-II/LDLR^-/-^ApoB^100/100 ^groups collapsed and died during isoflurane anesthesia.

### Cardiac reserve is preserved but reduced in atherosclerotic mice

To examine the myocardial viability, dobutamine stress echocardiography was performed to evaluate cardiac reserve. The timepoint of individual peak response after dobutamine administration varied between 5 and 15 minutes and analyses were performed from the maximal response values (Table 2). Dobutamine-induced chronotropic responses correlated inversely with baseline heart rates: C57Bl/6J mice with the lowest resting heart rate showed the highest increase of 13.2 ± 8.5% (*P *= 0.043 compared to baseline), whereas in IGF-II/LDLR^-/-^ApoB^100/100 ^mice the heart rate increased 9.9 ± 4.9% (*P *= 0.011) and in LDLR^-/-^ApoB^100/100 ^mice with the highest resting heart rate the increase was only 6.2 ± 6.5% (*P *= 0.070).

**Table 2:** Dobutamine stress induced changes in echocardiographic parameters.

%	C57Bl/6J	LDLR^-/-^ApoB^100/100^	IGF-II/LDLR^-/-^ApoB^100/100^
ΔHR	13.22 ± 8.45 (0.043)	6.19 ± 6.52 (0.070)	9.86 ± 4.93 (0.011)

ΔEF	14.87 ± 4.51 (0.001)	16.83 ± 1.52 (< 0.0001)	16.79 ± 8.53 (0.001)

ΔFS	15.48 ± 6.00 (0.005)	11.91 ± 2.18 (0.003)	13.74 ± 7.44 (0.0145)

ΔLVID_ED_	-9.4 ± 4.5 (0.009)	-12.7 ± 6.7 (0.016)	-9.3 ± 3.7 (0.032)

ΔLVID_ES_	-31.9 ± 11.1 (0.002)	-24.6 ± 6.8 (0.001)	-24.9 ± 13.0 (0.017)

ΔLVPW;d	18.0 ± 2.9 (0.099)	19.6 ± 8.4 (0.006)	18.5 ± 11.3 (0.021)

ΔLVPW;s	37.4 ± 19.1 (0.184)	29.7 ± 7.9 (0.0004)	28.3 ± 13.9 (0.004)

ΔLVAW;d	26.7 ± 22.0 (0.863)	23.0 ± 25.7 (0.078)	28.0 ± 12.0 (0.005)

ΔLVAW;s	32.3 ± 10.9 (0.001)	26.6 ± 15.6 (0.010)	28.0 ± 16.6 (0.004)

ΔLV Vol;d	-20.5 ± 9.6 (0.009)	-25.5 ± 14.1 (0.012)	-20.7 ± 7.6 (0.006)

ΔLV Vol;s	-61.1 ± 16.8 (0.002)	-50.0 ± 12.0 (0.002)	-51.6 ± 19.2 (0.012)

Values denote mean ± SD. Dobutamine challenge was performed to 5 (LDLR^-/-^ApoB^100/100 ^and IGF-II/LDLR^-/-^ApoB^100/100^) or 6 mice (C57Bl/6J) per strain. *P*-values from paired t-test comparing group baseline and the maximal response after dobutamine are shown in parenthesis.			

**Figure 4:**
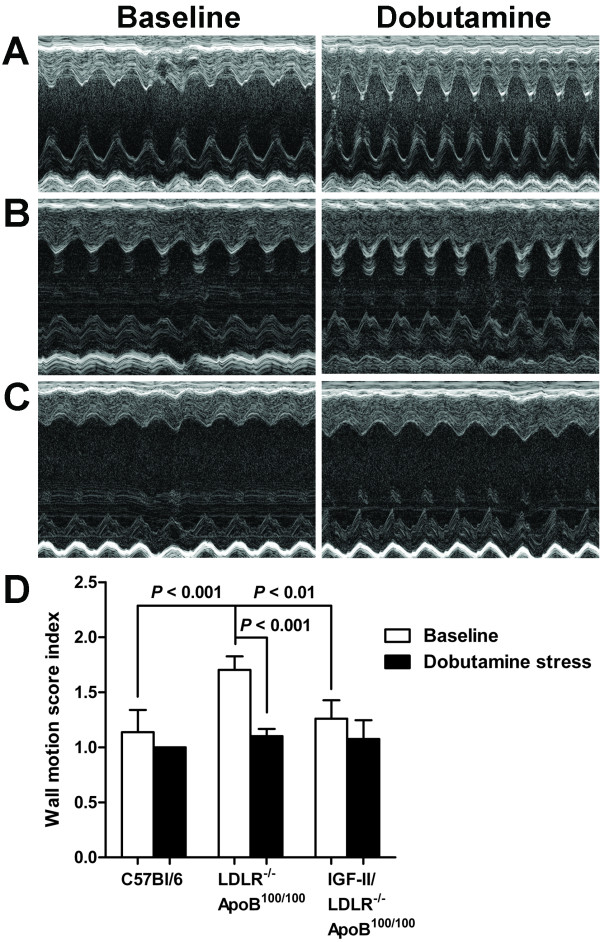
**Dobutamine stress echocardiography**. M-mode short-axis views of the left mid-ventricle before and after β-adrenergic stimulation with dobutamine in an 18-month-old A) C57Bl/6J, B) IGF-II/LDLR^-/-^ApoB^100/100 ^and C) LDLR^-/-^ApoB^100/100 ^mouse. Compared to the C57Bl/6J control, IGF-II/LDLR^-/-^ApoB^100/100 ^and LDLR^-/-^ApoB^100/100 ^mice show reduced contractility. D) Regional wall motion analysis represents improvement of systolic function after dobutamine in all mouse strains, although statistically significant only in LDLR^-/-^ApoB^100/100 ^mice. Results are mean ± SD of 5 (LDLR^-/-^ApoB^100/100 ^and IGF-II/LDLR^-/-^ApoB^100/100^) or 6 animals (C57Bl/6J).

In parallel, significant enhancement in systolic function (ΔEF, ΔFS) was seen in all strains and increased myocardial contractility, depicted by increased thickness of the LV walls, was as well observed (Table 2 Figure 4). Furthermore, both diastolic and systolic LV dimensions decreased significantly. Although rather equal in percentage, the overall response to dobutamine was diminished in relation to baseline values in both diabetic and non-diabetic LDLR^-/-^ApoB^100/100 ^mice. However, all mice responded to the β-adrenergic stimulation and their wall motion improved (Figure 4). Due to a limited sample size (n = 5-6/strain), change in the wall motion score index was statistically significant only in LDLR^-/-^ApoB^100/100 ^mice (*P *< 0.001). This was possibly influenced also by their significantly poorer baseline score (1.70 ± 0.12) compared to IGF-II/LDLR^-/-^ApoB^100/100 ^(1.26 ± 0.17, *P *< 0.01) and C57Bl/6J mice (1.14 ± 0.20, *P *< 0.001).

### Myocardial perfusion correlates with the atherosclerotic burden

Measured with contrast pulse sequence ultrasound, there were no statistically significant differences in LVAW perfusion between the groups (Table 1). However, a trend towards decreased myocardial perfusion was seen in LDLR^-/-^ApoB^100/100 ^and IGF-II/LDLR^-/-^ApoB^100/100 ^mice.

### Ultrastructural analysis of myocardium reveals degenerative changes in hypercholesterolemic mice

**Figure 5:**
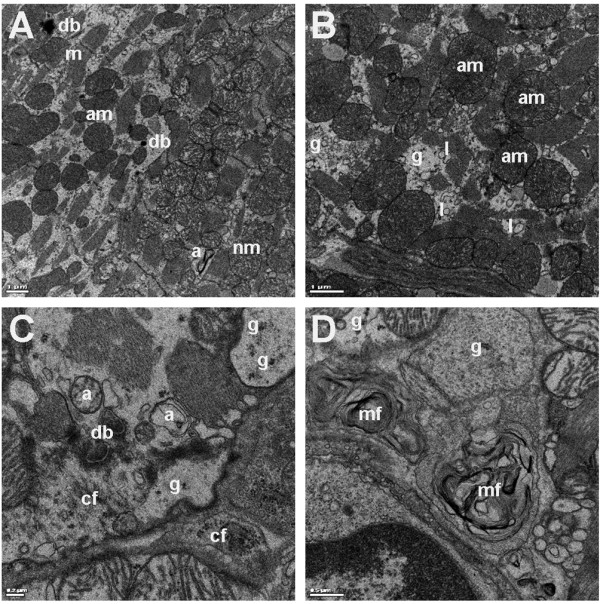
**Representative images of ultrastructural changes found in LDLR^-/-^ApoB^100/100 ^and IGF-II/LDLR^-/-^ApoB^100/100 ^mice**. A) On the left, impaired cytoarchitecture with increased interfibrillar space, dense bodies (db) and polymorphic and often doughnut-like mitochondria (am, abnormal mitochondria). Note autophagosome (a) in the preserved area. Nm, normal mitochondria; m, myofiber. B) Detail of the damaged area with increased number of enlarged abnormal mitochondria (am), lysosomes (l) and sparse glycogen deposits (g). C) Increased interfibrillar space filled with autophagosomes (a), dense bodies (db), collagen fibrils (cf) and glycogen deposits (g). D) Detail on myelin figures (mf) and glycogen deposits (g). Images are from IGF-II/LDLR^-/-^ApoB^100/100 ^mice. Bars 1.0 μm (A, B), 0.2 μm (C) and 0.5 μm (D).

In electron microscopy analysis ventricular myocardium showed degenerative changes (Figure 5). Cardiomyocytes with impaired cytoarchitecture, autophagosomes, decreased number of myofibrils and increased interfibrillar space with collagen fibrils, irregularly shaped dense bodies, myelin figures and focal deposits of glycogen were observed. Mitochondria were polymorphic and often doughnut-like. The changes were present in both LDLR^-/-^ApoB^100/100 ^and IGF-II/LDLR^-/-^ApoB^100/100 ^mice.

## Discussion

In the present study the effects of type 2 diabetes on coronary artery disease and cardiac function were investigated in 18-month-old mice after three months of Western diet. We found notable stenosis in proximal coronary arteries in both LDLR^-/-^ApoB^100/100 ^and diabetic IGF-II/LDLR^-/-^ApoB^100/100 ^mice and the stenoses were most severe in the left coronary artery, which was significantly narrowed in all animals of these groups despite compensatory vessel remodeling. Since type 2 diabetes increases significantly the risk of coronary events [2,25], we wanted to study if the cardiac outcome would be worse in the diabetic animals. Regardless of increased intimal calcification in aortic lesions of the IGF-II/LDLR^-/-^ApoB^100/100 ^mice, diabetes did not lead to a worsened CAD compared to hypercholesterolemic LDLR^-/-^ApoB^100/100 ^mice. Also LV function was equally impaired in both hypercholesterolemic mouse strains compared to age- and diet-matched C57Bl/6J mice. Based on these findings the impaired cardiac function in IGF-II/LDLR^-/-^ApoB^100/100 ^mice does not seem to be related to diabetes. This is likely subsequent to the high plasma cholesterol levels of the LDLR^-/-^ApoB^100/100 ^background in both models, since it is widely recognized that cholesterol is the main driving force behind atherogenesis in mice. However, because the cholesterol levels in our study were equal in diabetic and non-diabetic LDLR^-/-^ApoB^100/100 ^mice, detecting the effects of diabetes is possible, whereas the diabetes-induced elevation in plasma lipids seen in most models of diabetic atherosclerosis hampers dissecting the contribution of other metabolic factors on cardiovascular outcome [15].

Although genetically-modified mouse models of hypercholesterolemia are widely used in cardiovascular research [26,27], the status of coronary arteries and potential manifestation of myocardial infarction have been studied to a lesser extent. In the commonly used LDLR^-/- ^mice CAD seems to be absent even if atherogenic diet is used [28]. Lesions in coronary arteries and myocardial fibrosis were described in Western diet fed ApoE^-/- ^mice shortly after the model was generated [29], but since then controversial results have been reported: coronary artery occlusion and subsequent evidence of apical infarction are found by some [30], whereas in other studies the coronary arteries are found to be totally clean [31] or lesions are seen only in proximal parts of the arteries without signs of infracted myocardium [31-33]. The latter has been found also in the LDLR^-/-^ApoB^100/100 ^mice by Saraste et al. [34] and verified in the present study. In the severely hypercholesterolemic ApoE^-/-^LDLR^-/- ^mice both distal coronary artery lesions and myocardial infarction have been reported [35,36]. The gravest phenotype is in double knockout mice lacking scavenger receptor class B type I (SR-BI) and ApoE - the mice die of CAD induced myocardial infarction by 8 weeks of age [37].

Positive or outward arterial remodeling is a compensatory increase in vessel size in order to maintain luminal area in atherosclerosis [38]. Whereas clinical studies on vascular remodeling in different disease states are abundant, less is known about it in experimental models, especially mice. Positive remodeling has been observed in aortic sinus [39] as well as in carotid, thoracic and abdominal aortas of ApoE^-/- ^mice [24,40]. Consistent with this data, our results show enlargement of vessel areas in both aortic sinus and coronary arteries. In aortic sinus this compensatory remodeling resulted in maintenance of a lumen area equal to non-atherosclerotic C57Bl/6J controls, whereas in left coronary artery the lumen was significantly narrowed. Since stenosis was greater in coronary arteries (~80%) than in the aortic sinus (~60%), this might reflect a similar observation made in humans: once certain threshold in plaque deposition is exceeded, further enlargement might not be possible and lumen area is compromised by the growing lesion [38]. To our knowledge, vascular remodeling has been previously studied neither in mouse coronary arteries nor in a model of diabetic atherosclerosis. Because of the increased risk for cardiac events in diabetes, this is a matter of interest. Association between positive remodeling and unstable lesions has been suggested [41,42] and therefore it could be speculated that outward remodeling would be greater in diabetic patients with accelerated atherosclerosis and thus possibly also in the diabetic IGF-II/LDLR^-/-^ApoB^100/100 ^mice with a more inflammatory vascular phenotype and advanced lesions [20]. On the other hand, also negative remodeling could contribute to the increased incidence of cardiac events in diabetics. However, in the present study diabetes did not lead to either of these but the remodeling was equal compared to non-diabetic mice with similar atherosclerosis. Inconsistent findings have been done also in humans: both increased [43] and inadequate compensatory remodeling [44] have been detected with intravascular ultrasound, calling for more in-depth studies in experimental models.

Atherosclerosis as a progressive disease and aging is associated with general cardiovascular changes [45]. Compared to our former characterization of 15-month-old animals [20], older age of mice in the present study reflected as an analogous augmentation of atherosclerotic calcification in both diabetic and non-diabetic LDLR^-/-^ApoB^100/100 ^mice. Also plasma triglyceride levels of the IGF-II/LDLR^-/-^ApoB^100/100 ^mice were now higher than in LDLR^-/-^ApoB^100/100 ^mice and could in theory contribute to increased calcification. However, this is unlikely, since the level was still in the normal range and numerically only marginally higher than in the LDLR^-/-^ApoB^100/100 ^mice. In addition, in our previous study the situation was vice versa and yet there was more calcification in the IGF-II/LDLR^-/-^ApoB^100/100 ^mice. Thus the difference in plasma triglycerides most probably describes natural fluctuation. Moreover, no significant correlation between lipid levels and level of calcification were noted. Therefore the physiological significance of minor changes in triglycerides is unlikely, especially in the presence of severe hypercholesterolemia.

Despite prominent stenosis in proximal coronary arteries and observed regional akinesia in LVAW of LDLR^-/-^ApoB^100/100 ^and IGF-II/LDLR^-/-^ApoB^100/100 ^mice, signs of myocardial infarction were not found. Thus, the observed severe LV dysfunction most likely results from adaptation to chronic hypoperfusion rather than acute ischemic events. In support of this the resting perfusion in LVAW was only slightly reduced, which is in line with the study observing reduced coronary flow reserve in old, Western diet fed LDLR^-/-^ApoB^100/100 ^mice [34]. Cardiac functional reserve after dobutamine challenge was preserved, although depressed compared to C57Bl/6 controls, indicating that the dysfunctional myocardium of LDLR^-/-^ApoB^100/100 ^and IGF-II/LDLR^-/-^ApoB^100/100 ^mice was not scarred but viable. Dobutamine stress echocardiography is a feasible non-invasive method to gain information about cardiac function and capacity, and to predict reversibility of wall motion after revascularization [46]. In ApoE^-/- ^mice the cardiac functional reserve has been found to be reduced [47]. Also in diabetic db/db [48] and ob/ob mice [18] the response to β-adrenergic stimulation has been shown to be decreased. So far the only study done in a mouse model of diabetic atherosclerosis showed impaired cardiac reserve in the ob/ob/LDLR^-/- ^mice [18].

In patients with ischemic cardiomyopathy, hibernating myocardium is an important clinical entity with significant prognostic value when considering revascularization procedures. Myocardial hibernation is referred as a state of reduced contractile function in the setting of CAD that is reversible with revascularization. Interestingly, various results in the present study suggest myocardial hibernation: LV dysfunction distal to coronary stenosis, preserved inotropic reserve, absence of necrosis and minor reduction in myocardial perfusion. Also the ultrastructural findings in LDLR^-/-^ApoB^100/100 ^and IGF-II/LDLR^-/-^ApoB^100/100 ^mice support the possibility of hibernation, since the morphological hallmarks of chronic myocardial hibernation include signs of atrophy (especially contractile myofibrils and loss of cardiomyocytes) and degenerative changes (disorganization of cytoskeleton, cellular debris in interstitium, increased collagen and glycogen) without necrosis [49]. As a limitation to the hypothesis of myocardial hibernation, some of the changes seen in LDLR^-/-^ApoB^100/100 ^and IGF-II/LDLR^-/-^ApoB^100/100 ^mice could be induced also by aging. However, there is a general lack of suitable animal models that has hampered the studies of myocardial hibernation. Because achieving reproducible and chronic partial coronary artery stenosis without infarction is technically very challenging, most experimental studies of myocardial hibernation have been done in larger animals, such as dogs and pigs [49,50]. The few mouse models reported are mainly based a short-term hypoperfusion [51] or genetic manipulation of *e.g*. myocardial angiogenesis [52]. Since chronic CAD and the resulting gradual intravascular occlusion are the major causes of ischemic cardiomyopathy in humans, it would be aspired to have them manifested also in the experimental animal model. Even if the model would not perfectly reproduce the human condition and the actual revascularization procedures as an affirmation of the hibernation are not technically feasible in mice, animal models can provide unique and valuable information.

## Conclusions

Hypercholesterolemic LDLR^-/-^ApoB^100/100 ^mice with or without type 2 diabetes develop significant coronary artery atherosclerosis with positive arterial remodeling, severe left ventricular dysfunction with regional akinesia distal to coronary stenosis without signs of infarction, and represent preserved but reduced cardiac functional reserve and histological features referring to chronic myocardial hibernation. Hence, the cardiac outcome was not worsened by type 2 diabetes, probably because of dominant effect of severe hypercholesterolemia caused by the LDLR^-/-^ApoB^100/100 ^background. Since animal models describing type 2 diabetic macrovascular diseases are few, a thorough characterization of the vascular and cardiac phenotype of a mouse model combining common metabolic disorders, CAD and chronic myocardial hypoperfusion provides valuable information and offers a tool for experimental and translational research.

## Abbreviations

A: autophagosome; am: abnormal mitochondria; ANOVA: analysis of variance; Apo: apolipoprotein; AW: anterior wall; CAD: coronary artery disease; cf: collagen fibril; CPS: contrast pulse sequence; d: diastole; db: dense bodies; ED: end-diastole; EF: ejection fraction; EKV: electrocardiogram kilohertz-based visualization; ES: end-systole; FS: fractional shortening; g: glycogen; GTT: glucose tolerance test; ID: internal diameter; IGF-II: insulin-like growth factor-II; l: lysosome; LAX: lateral axis; LDLR: low density lipoprotein receptor; LV: left ventricle; m: myofiber; mf: myelin figures; MI: myocardial infarction; nm: normal mitochondria; ND: not detected; PW: posterior wall; s: systole; SAX: sagittal axis; SD: standard deviation; SR-BI: scavenger receptor class B type I; Vol: volume.

## Competing interests

The authors declare that they have no competing interests.

## Authors' contributions

SEH conceived and designed the study, participated in echocardiography and perfusion studies, carried out histological, morphometrical and glucose metabolism analyses, performed statistical analyses and drafted the manuscript. MM participated in echocardiography and perfusion studies, and helped draft the manuscript. MH contributed in cardiac function analyses and interpretation, and helped draft the manuscript. PIM performed analysis of plasma lipids. EL participated in histological analyses and in vivo studies. IK performed electron microscopy analysis and helped draft the manuscript. FB and ML participated in study design and helped to draft the manuscript. SYH helped conceive the study, participated in its design and interpretation, and helped draft the manuscript. All authors read and approved the final manuscript.

## Supplementary Material

Additional file 1 **Long axis EKV™ cine loop of a C57Bl/6J mouse**. Transthoracic echocardiography in C57Bl/6J mouse was performed with Vevo 770 high-resolution imaging system. Long axis cine loops showed preserved normal function of the left ventricular walls. No atherosclerotic lesion development was seen in either aorta or coronary arteries. Ejection fraction was 79.9%. Click here for file

Additional file 2 **Long axis EKV™ cine loop of a LDLR^-/-^ApoB^100/100 ^mouse**. Transthoracic echocardiography was performed with Vevo 770 high-resolution imaging system. Long axis electrocardiogram kilohertz-based visualization (EKV™) cine loops show a poor systolic function (ejection fraction 18.1%) in a LDLR^-/-^ApoB^100/100 ^mouse. The left ventricle was dilated with anteroapical akinesia and hypokinetic posterior wall. The mouse had aortic cross-sectional lesion area of 73.3% with calcification and the left coronary artery was markedly stenosed (94.3%). Click here for file

Additional file 3 **Long axis EKV™ cine loop of an IGF-II/LDLR^-/-^ApoB^100/100 ^mouse**. Transthoracic echocardiography was performed with Vevo 770 high-resolution imaging system. Long axis cine loops showed markedly reduced anteroapical wall motion in the left ventricle in an IGF-II/LDLR^-/-^ApoB^100/100 ^mouse, which presented calcified lesions covering 70% of the aortic lumen area at the sinus level and significant stenosis in both left and right coronary arteries (99.5% and 93.2%, respectively). Ejection fraction was reduced (41.2%) compared to control C57Bl/6 mice. Click here for file
